# A shape tailored gold-conductive polymer nanocomposite as a transparent electrode with extraordinary insensitivity to volatile organic compounds (VOCs)

**DOI:** 10.1038/srep33895

**Published:** 2016-09-22

**Authors:** Rania Khalil, Shahin Homaeigohar, Dietrich Häußler, Mady Elbahri

**Affiliations:** 1 Nanochemistry and Nanoengineering, Faculty of Engineering, University of Kiel, Institute for Materials Science, Kaiserstrasse 2, 24143 Kiel, Germany; 2 Physics Department, Faculty of Science, Zagazig University 44519, Zagazig, Egypt; 3 Nanochemistry and Nanoengineering, School of Chemical Technology, Department of Chemistry and Materials Science and Engineering, Aalto University, Kemistintie 1, 00076 Aalto, Finland; 4 Faculty of Engineering, University of Kiel, Institute for Materials Science, Synthesis and Real Structure Group, Kaiserstrasse 2,24143 Kiel, Germany; 5 Nanochemistry and Nanoengineering, Helmholtz-Zentrum Geesthacht, Max-Planck-Str.1, 21502, Geesthacht, Germany

## Abstract

In this study, the transparent conducting polymer of poly (3,4-ethylenendioxythiophene): poly(styrene sulphonate) (PEDOT:PSS) was nanohybridized via inclusion of gold nanofillers including nanospheres (NSs) and nanorods (NRs). Such nanocomposite thin films offer not only more optimum conductivity than the pristine polymer but also excellent resistivity against volatile organic compounds (VOCs). Interestingly, such amazing properties are achieved in the diluted regimes of the nanofillers and depend on the characteristics of the interfacial region of the polymer and nanofillers, i.e. the aspect ratio of the latter component. Accordingly, a shape dependent response is made that is more desirable in case of using the Au nanorods with a much larger aspect ratio than their nanosphere counterparts. This transparent nanocomposite thin film with an optimized conductivity and very low sensitivity to organic gases is undoubtedly a promising candidate material for the touch screen panel production industry. Considering PEDOT as a known material for integrated electrodes in energy saving applications, we believe that our strategy might be an important progress in the field.

Nowadays, to make communicative technology portable and user friendly, we need to miniaturize electronic devices as smart phones, tablets etc.^1^. In this regard, touch screen panels as a user interface technology have successfully minimized the size of communicative and multimedia devices. For an efficient performance, these panels should have an optimum transmittance, conductivity, resolution, resistance to any surface contamination, durability and display size^1^,^2^. These characteristics are mainly dependent on the material, they are composed of. Conventionally, such transparent electrodes are made of indium-tin oxide (ITO) due to optimum electrical conductivity and optical transparency^3^. However, shortcomings such as high price of the indium source, depletion of ITO and its brittleness as well as high processing temperature for production of ITO have led to emergence of novel conductive flexible polymer thin films as potential candidates^1^,^3^,^4^,^5^,^6^,^7^,^8^,^9^. Thanks to excellent electrical and optical properties, PEDOT:PSS has been nominated as a promising transparent electrode material^10^,^11^. PEDOT:PSS has drawn attention due to its suitable electrical conductivity, low band gap and excellent environmental stability^8^,^12^. However, despite various pros of PEDOT:PSS, its conductivity is not as high as ITO’s^1^. Additionally, similar to other conducting polymers, the oxidation level of the polymer is readily influenced by chemical or electrochemical doping/de-doping (oxidation/reduction) mechanisms. This effect causes a sensitive and quick response to particular chemical substances and loss of electrical conductivity^12^. For instance, when exposed to VOCs, commonly found in indoor and outdoor air, a decline of conductivity occurs. This is why, PEDOT:PSS^13^,^14^,^15^,^16^ and the nanocomposites made thereof^17^,^18^,^19^,^20^ have shown a high potential as gas sensors.

Accordingly, to employ PEDOT:PSS as touch panels in advanced portable electrical devices, it is vital to optimize electrical conductivity while minimizing its sensitivity to VOCs. Here, to fulfill such requirements, we dope PEDOT: PSS by inclusion of gold (Au) nanoelements (NEs) in different morphologies i.e. nanospheres (NSs) and nanorods (NRs). In our belief, diversity of the nanofillers’ shape/aspect ratio, thereby width of the interfacial region, can play an important role in tuning sensing, optical and electrical properties of the PEDOT:PSS. Moreover, a low filling factor of the nanofillers is intentionally envisaged to maintain optimum transparency of the polymer. To the best of our knowledge, no report in the literature describes a similar approach for minimizing VOC sensitivity while augmenting conductivity of PEDOT: PSS.

## Results and Discussion

**Figure 1:**
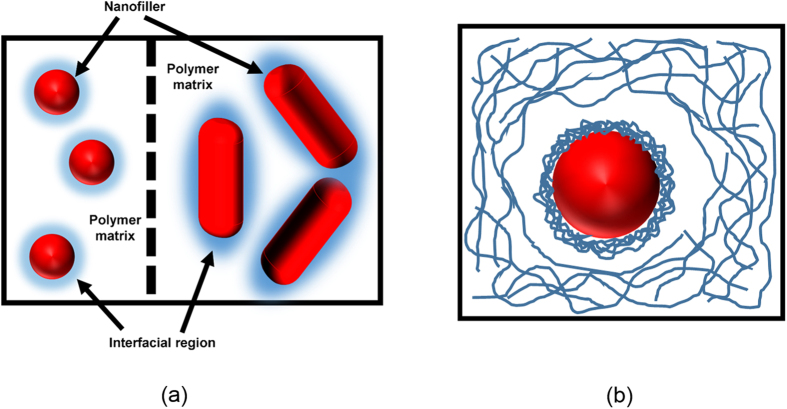
Schematic of the nanocomposite’s structure. (**a**) the interfacial region between the nanofillers and polymeric matrix enhances the macroscopic polarizability of the composites; (**b**) the polymer chains’ deformation around the nanofillers leads to their severe entanglement at this area.

Embedding of metal nanoparticles into polymeric hosts is of the fundamental routes for creation of novel nanocomposites with unique applications in modern technology^21^,^22^,^23^,^24^,^25^,^26^,^27^,^28^,^29^. In this regard, macroscopic properties (electrical, optical, mechanical etc.) of nanocomposites can be tailored by changing the volume fraction of the nanofillers. For instance, electrical properties of the conducting transparent polymers is strongly improved by inclusion of conductive nanofillers, but at a high filling factor^30^,^31^. In contrary, here, for the first time, we aim to benefit from incorporation of metal nanofillers at a very low filling factor (~5%) in two directions of optimization of electrical conductivity and more importantly minimizing VOCs sensitivity of the material without compromising its transparency. Our concept relies on the role of interfacial region (Fig. 1a) between the nanofillers and polymeric host which would enhance the macroscopic polarizability of their respective composites, thereby optimizing the relevant (shape dependent) structural properties.

While Clausius-Mossotti has described composites as homogeneous media, which is macroscopically acceptable for a non-polar medium, the role of the polar surrounding (host) medium has to be considered^32^. For instance, by incorporating metal nanoparticles into a conductive polymer, the dipolar coupling at the interface increases the mutual polarization, hence affects the macroscopic response (i.e. electrical conductivity). On the other hand, as shown in Fig. 1b, the polymer chains’ deformation around the nanofillers (due to polar-polar affinity) leads to their severe entanglement at this area, thereby optimization of the structural properties such as mechanical ones^33^,^34^,^35^,^36^,^37^.

Postulating the interfacial region as an important factor in structural properties of the nanocomposites, geometry of the nanofiller i.e. its aspect ratio can certainly play an important role in design of the nanocomposites with tuned properties. For instance, an evolution from spherical to rod shaped particles increases the interfacial region and can ultimately tailor the properties even in a diluted nanoparticle regime.

**Figure 2:**
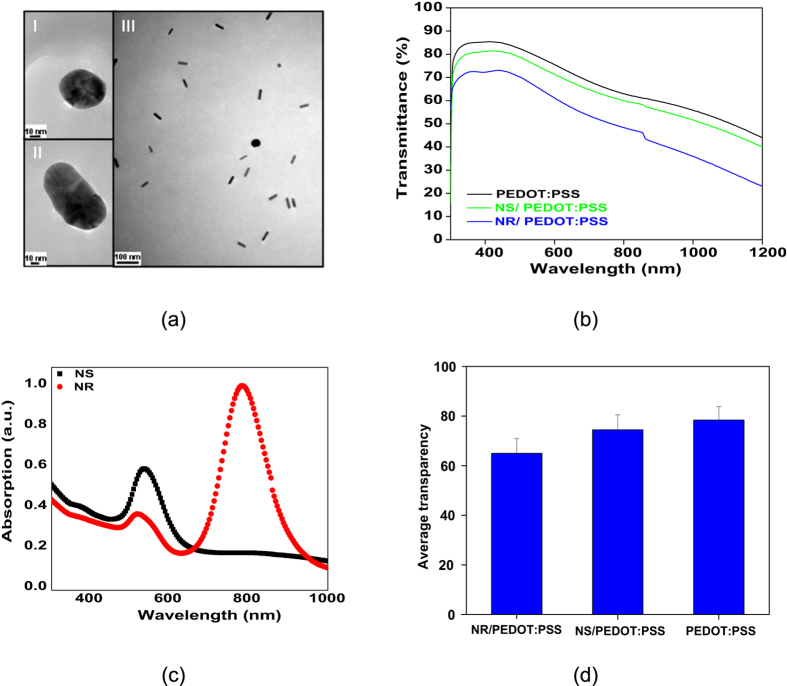
Morphology and optical properties of the Au NEs and their respective nanocomposites. (**a**) TEM images of the gold NSs (I) and NRs (II) synthesised and a typical example of the NRs blended in polymeric solutions homogenously with a volume fraction of ~5% ; (**b**) The UV-vis transmittance spectra of the pristine PEDOT:PSS, Au NS/PEDOT:PSS and Au NR/PEDOT:PSS; (**c**) The UV-vis absorption spectra of the Au NEs within their respective aqueous solutions; and (**d**) average transparency of the pristine PEDOT:PSS, Au NS/PEDOT:PSS and Au NR/PEDOT:PSS.

Based on this concept, in our study, gold NSs and NRs were synthesised through the standard methods mentioned in the experimental part and then blended in polymeric solutions homogenously with a weight percent of 5% (Fig. 2aI-III). The main reason for use of such a low filler content was maintaining the polymer’s transparency while optimizing its structural properties. Figure 2b shows the transmittance spectrum of the pristine and nanocomposite samples of PEDOT:PSS. As seen here, compared to the pristine PEDOT:PSS, the nanocomposites exhibit minimal loss of transparency in the visible range. The average transmittances of pristine and Au NS-NR/PEDOT:PSS are ~77, 75 and 69%, respectively. The magnitude of this loss is completely dependent on absorption and scattering contributions of the plasmonic nanoparticles. In this regard, scattering effect of the nanorods is more notable, and there is a linear relationship between absorption and aspect ratio of Au NEs^38^, thus lowering the transparency up to 10%. In this regard, UV-Vis absorption spectra of the samples were employed as evidences of effect of the morphology of the Au NEs on absorption behavior of the samples. In general, noble metal nanospheres are able to show a strong UV-Vis absorption band when the incident photon frequency resonates with the collective excitation of the conduction electrons. This resonance is called as “localized surface plasmon resonance” (LSPR)^39^. As seen in Fig. 2c, a single absorption peak appears at 544 nm in the spectrum that is attributed to formation of Au NSs and their transverse surface plasmon resonance^40^. On the other hand, Au NRs absorb in the near IR region of the electromagnetic spectrum and show two well-separated absorption bands^41^. While the appearance of the first band at around 520 nm i.e. in the blue-green region is due to the transverse surface plasmon vibration, the appearance of the second band in the red-near infrared region is attributed to the longitudinal surface plasmon absorption^40^,^42^,^43^. Nevertheless, as seen in Fig. 2d, the average transparency of the conductive polymer is not indeed sacrificed by the adopted nanocomposite strategy.

**Figure 3:**
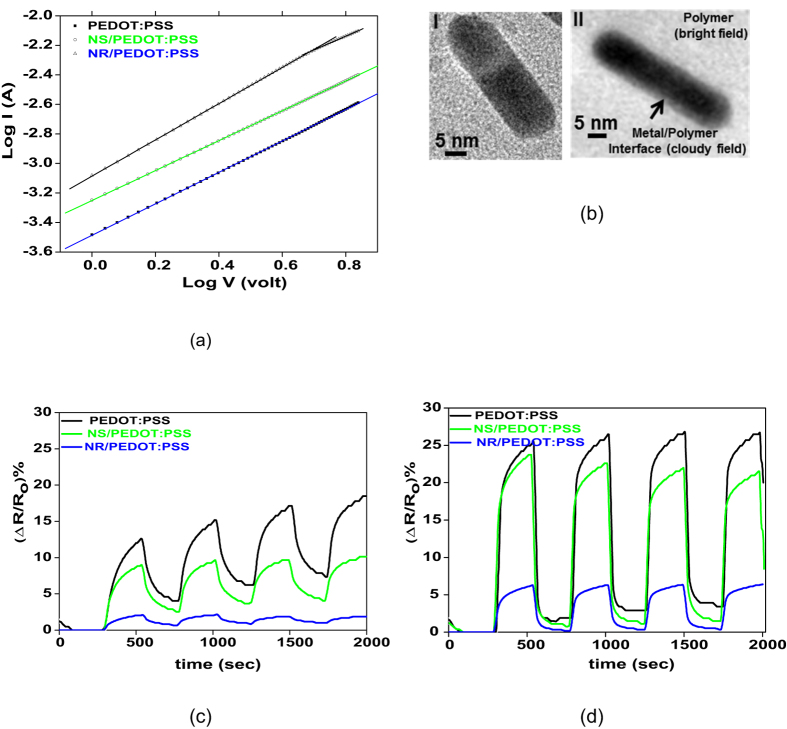
Conductivity and VOC sensitivity. (**a**) *I-V* Characteristics of pristine and nanocomposite samples of PEDOT:PSS; (**b**) TEM image of pure Au NR (I) versus that of the polymer embedded Au NR (II), clearly showing the interfacial region as a distinct contrast between the dense polymeric chains located on the nanorods (cloudy region) and the rest of the polymer (bright region); resistance response of PEDOT:PSS and its derivative nanocomposites to (**c**) toluene and (**d**) ethanol.

Influenced by polarizability of the interfacial region and its intensity depending on the aspect ratio of the nanofiller, we assume that the electrical properties of the nanocomposites should be superior to their pristine counterpart. Noteworthy, there is no distinct integrated pathway for electron transfer through the nanofillers due to their very limited content. Based on our assumption concerning the important role of aspect ratio of the nanofillers on the level of polarizability of the structure, among the nanocomposites, the most optimum electrical conductivity should be obtained for Au NR/PEDOT:PSS whose structure possesses a higher interfacial region and polarizability than its nanosphere based counterpart. In this regard, Fig. 3a shows *I-V* characteristics of PEDOT:PSS as pristine and nanocomposites at room temperature. The relationship between current (*I*) and voltage (*V*) was determined according to the following equation (1)^44^:


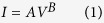


where *A* and *B* constants represent capability and property of electrical conduction, respectively. A logarithmic operation can be used to determine the constants as slope and intercept of log *I* Vs. log *V* linear graphs (equation (2)):


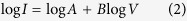


Based on this figure, *A* and *B* constants were calculated and tabulated in Table 1. This table shows that the *B* value for all the samples as pristine and nanocomposite is nearly 1. Thus, the samples exhibit an ohmic behavior across the applied voltage range^44^. Moreover, based on the *A* values, representing the electrical conductance, conductivity of the pristine PEDOT:PSS is optimally improved by inclusion of the Au NEs. Quantitatively, electrical conductivity of the samples can be determined using the equation (3)^45^,^46^:


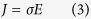


where *J* is the current density (*μA/cm*^2^), *σ* is the electrical conductivity (*S*/*cm*) and *E* is the applied electrical field (*V*/*μm*). The values of electrical conductivity as presented in Table 1 again imply enhancement of the conductivity of the pristine PEDOT:PSS after nanohybridization. In general, in the conducting polymers, permanent dipoles are replaced by strong charge (polaron and bipolaron) trapping centers^47^,^48^. The localized motion of such centers acts as an electric dipole when subjected to an applied external electric field^49^. The electric field results in hopping of the localized charge carriers to neighboring sites, thereby occurrence of the dielectric relaxations. The charge hopping creates a continuous network enabling the charges flow throughout the sample i.e. forming electrical conduction. In case of lack or absence of strong charge trapping centers, the charge hopping is developed across the sample, thereby a continuous current flows at low frequencies. When the Au nanoclusters are incorporated within PEDOT:PSS, the charge trapping centers are minimized and a coupled dipolar response in the interfacial region is created by the applied field. Considering a larger interfacial region in the NR/PEDOT:PSS system than its nanosphere based counterpart, proportionally the conductivity has a higher increasing trend in such a system.

Existence of the interfacial region was proved by TEM. As seen in Fig. 3b, there is a distinct contrast between the dense polymeric chains located on the nanorods and the rest of the polymer. Whereas as previously shown in Fig. 2a, such interface can by no means seen in the case of suspended nanorods deposited on a carbon grid. The bonding between the Au nanoelements and sulphur atom of PEDOT is thought to be the reason behind formation of an integrated and dense interface and as a result it can lead to optimization of the structural properties e.g. thermal properties of the nanocomposites, in addition to electrical ones. TGA results, as shown in Table 2, imply enhancement of thermal decomposition temperature as well as residual mass i.e. thermal stability of the nanocomposites compared to the pristine sample. The effect is more notable regarding the Au NR/PEDOT:PSS, mainly due to the high aspect ratio of the NRs and thus their larger interfacial region. The enhanced thermal stability of the nanocomposites is attributed to the reduced mobility of the PEDOT:PSS chains when bound to the Au nanofillers, acting as physical barriers for the polymer network.

The VOCs are of the main environmental pollutants commonly found in the soil, environment, and atmosphere^18^. Hence, it is very realistic to consider interaction of VOCs with conductive touch screen panels and thereby deteriorating their performance. As mentioned earlier absorption of VOCs into PEDOT:PSS can lead to loss of conductivity. Hence, here, we attempted to minimize sensitivity of the polymer to VOCs via inclusion of Au nanofillers. To prove applicability of this idea, the responses of pristine PEDOT:PSS and Au NEs/PEDOT:PSS to VOCs were investigated by measuring the change in their electrical resistance.

Investigation of the electrical properties of the pristine and nanocomposite PEDOT:PSS samples upon exposure to polar and non-polar VOCs of ethanol and toluene, respectively, reveals that the nanoparticles have profound effects on immobility of the host polymer chains and restrict diffusion of VOC molecules, thus loss of conductivity i.e. sensitivity.

As seen in Fig. 3c,d, the response magnitude of the pristine PEDOT:PSS to all the VOCs is always higher than that of its nanocomposite counterparts especially Au NR/PEDOT:PSS. It is assumed that the Au NRs and their formed dense interfacial region with the polymer can act as a robust physical barrier against penetration of the VOC molecules and lower disintegration of conduction path of the polymer by the VOC induced swelling. Thus, this nanocomposite is very slightly sensitive to the VOCs studied. The sensitivity of Au NR/PEDOT:PSS to ethanol (166 ppm) and toluene (150 ppm), at ambient temperature is only 5 and 1.5%, respectively. To comprehend the sensitivity level of our system, a literature survey was performed. The survey could prove that similar studies based on PEDOT:PSS nanocomposites as VOC resistant transparent electrodes are very rare and our system is very unique in terms of transparency and conductivity while negligible VOC sensitivity. This structure owes its amazing properties to its critically low content of fillers with high aspect ratio thus interfacial region. Interestingly, the similar systems in terms of matrix composition i.e. PEDOT:PSS are mostly considered in an opposite direction as a gas sensor, implying susceptibility of the polymer to interact with organic gases^13^,^19^,^50^,^51^,^52^,^53^,^54^. In this category, a comparable negligible sensitivity to ethanol and toluene has also been stressed by Dehsari *et al*.^51^ for a nanocomposite system of Copper(II) phthalocyanine supported on a 3dimensional nitrogen-doped graphene (CuTSPc@3D-(N)GF)/PEDOT:PSS. This material can show a sensitivity of only 5.5 and 2.3% to ethanol and toluene (both 200 ppm) at ambient temperature, respectively^51^. However, this system has only the potential of a gas sensor and is unable to offer transparency due to the nature of the filler and its structure. As seen in Fig. 3c,d, the sensitivity values for the pristine and Au NS/ PEDOT:PSS are larger and over 23 and 8% for ethanol and toluene, respectively. This means, upon exposure of our PEDOT:PSS sample to ethanol, its resistance is enhanced. While, owing to the high affinity of polar ethanol to hydrophilic PSS, conductivity could even rise through re-arrangement of PSS and PEDOT domains. In fact, when subject to ethanol, the Coulomb interaction between positively charged PEDOT and negatively charged PSS dopants via a screening effect between counter ions and charge carriers decreases. This conformation change, leading to formation of separate PSS and PEDOT crystallites and reduction of their respective π-π stacking distances (stronger interchain coupling) optimizes the hopping rate and conductivity^8^,^9^,^13^. The reason behind discrepancy in sensing behaviour of our PEDOT:PSS sample with such assumption should be sought in the film formation conditions. During the spin coating process, the polymer is rapidly dried, thus morphology is far from thermodynamic equilibrium state^55^,^56^. In such a case, inclusion of ethanol can only scatter randomly arranged PEDOT domains and enlarge their interchain distance (swelling), thus lowering conductivity.

Interestingly, the resistance of Au NR/PEDOT:PSS almost instantaneously recovers to the original level when the VOC exposure is terminated and the cycle test could be performed more than three times. In contrary, the pristine and Au NS/PEDOT:PSS do not show such reversibility of resistance. The recovery time of Au NR-NS/PEDOT:PSS for ethanol and toluene are 240 and 230s, respectively. The recovery of the resistance once again implies negligible penetration of the VOC molecules into the nanocomposite structure.

As mentioned earlier, the rise of electrical resistance of PEDOT:PSS and Au/PEDOT:PSS when subjected to organic gases can be attributed to the diffusion of VOC molecules into the PEDOT:PSS structure and subsequently swelling that can destroy the conduction pathway^12^,^18^. The eventual swelling of the polymer by interaction with VOCs can be proved by consideration of the Flory-Huggins interaction parameter (equation 4)^18^:


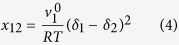


where 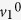 is the molar volume of the solvent (*cm*^3^/*mol*), *T* is the temperature (*K*), *R* is the ideal gas constant (*J*/*K.mol*), *δ*_*1*_ is the solubility parameter of the solvent (*J*^*1/2*^/*cm*^*3/2*^), and *δ*_*2*_ is the solubility parameter of the polymer (*J*^*1/2*^/*cm*^*3/2*^). The solubility parameter of PEDOT and PSS components were considered individually and the interaction parameter of the solvents with each one was determined.

An optimum interaction of polymer and solvent is obtained for low or null values of χ_12_. In Table 3, the calculated values of the interaction parameters for the different solvent/polymer systems are tabulated.

As seen in Table 3, *χ*_*12*_ of ethanol/PEDOT is slightly larger than that of toluene/PEDOT. This fact implies a slightly higher interaction tendency between toluene and PEDOT compared to ethanol and PEDOT. In contrary, this value for ethanol/PSS is significantly smaller than that of toluene/PSS. Therefore, a higher response magnitude of the polymer blend towards ethanol is seen (Fig. 3d). Such interaction tendency of the VOCs to different parts of the polymer blend is most likely related to the chemical functionality and polarity of them. In this regard, also physical factors of molar volume and concentration of the VOCs (ppm), can play a role. As shown in Table 3, the lower molar volume of ethanol than that of toluene facilitates its penetration into the polymer. Moreover, the ppm concentration of VOC can contribute to the response magnitude of the polymer towards the VOCs. This quantity in a conical flask placed in a glove box was calculated at room temperature via the following equation (5)^18^:


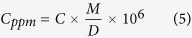


where *C*, *M* and *D* are the concentration, molecular weight (*g*/*mol*) and density (*g*/*cm*^3^) of the VOC, respectively. In case of envisaging VOC as a saturated ideal gas, its concentration can be estimated through the following equation (6)^18^:


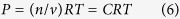


where *R* is the ideal gas constant and *P* is the saturated vapor pressure (*kPa*). Table 3 shows that the ppm concentration of ethanol is higher than that of toluene. This implies a larger number of ethanol molecules are present in a conical flask at room temperature in contact with the samples and thereby involved in the swelling process. Accordingly, the resistance of the pristine and nanocomposite samples of PEDOT:PSS shows a higher response magnitude towards ethanol.

**Figure 4:**
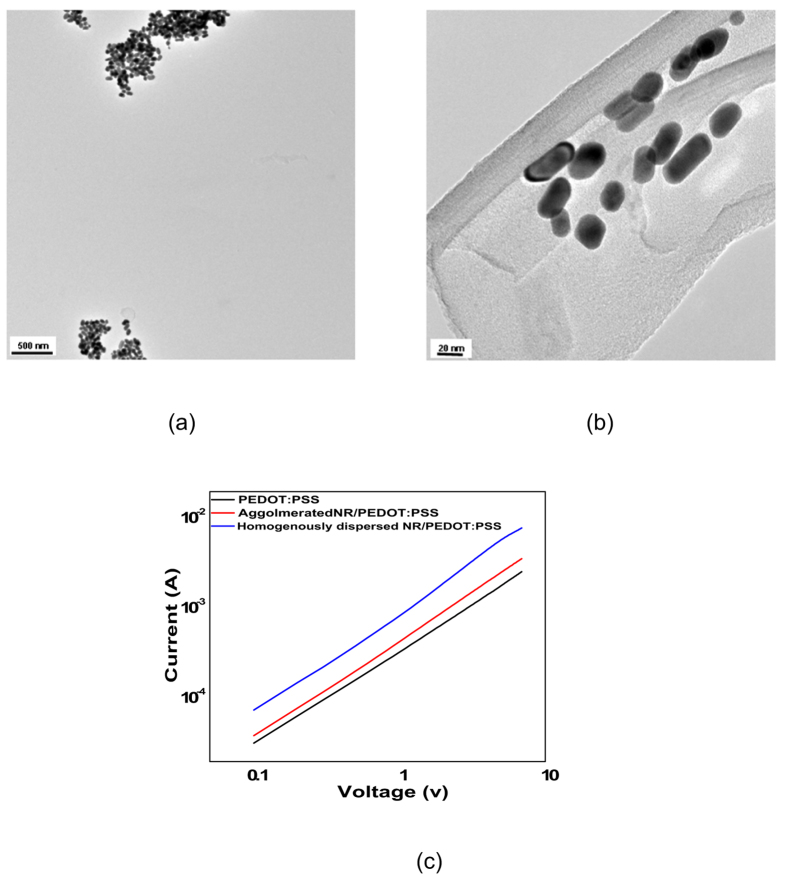
Agglomeration of Au NRs and its effect on conductivity. (**a**,**b**) agglomeration of Au NRs illustrated in TEM images with different magnifications; (**c**) The effect of agglomeration of Au NRs on conductivity of their respective nanocomposites.

To develop a transparent conductive and at the same time gas resistant nanocomposite, the fillers should be homogenously dispersed. This important requirement necessitates employment of the fillers as little as possible to prevent eventual agglomeration. When 5 wt% of the Au NRs, for instance, were used an optimum combination of conductivity, transparency (despite a 10% loss) and low gas sensitivity was obtained mainly due to a homogenous distribution of the nanofiller. But, as seen in Fig. 4a–c and based on the represented values in Table 4, when the filling factor slightly increased, due to agglomeration of the nanorods induced by eventual rise of the solution viscosity, conductivity enhancement was less significant. The reason is undoubtedly intimate contact of the nanorods, thus loss of interfacial region and its polarizability. This is the case for the VOC sensitivity, as well. In case of agglomeration at a higher filling factor, loss of the interfacial region leads to a lower hindrance against penetration of the VOCs, thus rise of resistance of the sample i.e. a higher gas sensitivity. Accordingly, through this study, 5 wt% is determined as the threshold filling factor, over which properties decline due to a severe agglomeration, leading to loss of the interfacial region.

## Conclusion

Interaction of organic vapors present everywhere with transparent conductive polymers leads to loss of their conductivity. This problem has ever been a big challenge ahead of touch screen panels production industry. Hence, finding solutions to address such a shortcoming has been always a research priority in the relevant industry. In this regard, we adopted a nanohybridization strategy, not only to optimize electrical conductivity of a well-known transparent conductive polymer of PEDOT:PSS, but also to minimize its sensitivity to volatile organic compounds. The results were promising in terms of conductivity as well as insensitivity to VOCs. In addition, this approach did not sacrifice transparency of the PEDOT:PSS thin films. Our approach i.e. nanohybridization of PEDOT:PSS by inclusion of gold nanorods holds great promise for development of advanced novel touch screen panels with desirable durability.

## Methods

PEDOT:PSS as pellet (ORGACON^TM^ Dry, Batch No. A650000AD) was supplied from Agfa (Belgium). The chemicals used for synthesis of gold NEs including HAuCl_4_, Sodium citrate tribasic dehydrate, L-Ascorbic acid, Silver Nitrate (AgNO_3_) and Hexadecyltrimethylammoniumbromide (CTAB, C_19_H_42_BrN) were all purchased from Sigma - Aldrich Co. (USA). Additionally, Sodium borohyride (NaBH_4_ 99%) was obtained from Fluka (Switzerland). All the chemicals were used as received.

The Au NSs were prepared via the sodium citrate reduction method as reported by Bi *et al*.^59^. In this method, 100 ml HAuCl_4_ solution (0.01%) was heated at 100 °C under continuous stirring. Then, 0.7 ml 1% sodium citrate solution was added. The entire solution was still stirred and heated at 100 °C for 10 min. Afterwards, the heating process stopped and the solution was stirred for an additional 15 min and then cooled down to room temperature. At last, the gold colloid solution was stored at 4 °C in dark bottles.

The Au NRs were produced via the seed-mediated growth method^60^. In this technique, the seed and growth solutions were made as follows:

*The seed solution*: CTAB solution (5 ml, 0.2 M) was mixed with 5 ml 0.5 mM HAuCl_4_. While stirring the solution for 2 min, 0.6 ml ice-cold of 0.01 M NaBH_4_ was added. Consequently, the color of the solution transformed to brownish yellow. The resultant solution was stored at 25 °C.

*The growth step*: CTAB (5 ml, 0.2 M) was added to 0.27 ml 0.004 M AgNO_3_ solution at 25 °C. Next, 5 ml 1 mM HAuCl_4_ was added to the resultant solution, and after a while gentle stirring, 70 μl 0.0788 M ascorbic acid was added. Ascorbic acid as a mild reducing agent decolorizes the dark yellow growth solution.

Ultimately, 12 μl of the seed solution was added to the growth solution at 27–30 °C. The color of the solution gradually changed after 10–20 min. Upon this visual change, the solution was centrifuged several times. The resultant precipitate was subsequently added to 12 ml de-ionized water.

An aqueous mixture of PEDOT:PSS and Au NEs was made through mixing an aqueous solution of the polymer (0.005 gm in 0.15 ml deionized water) and an aqueous suspension of Au NEs (2.85 ml). The mixture composition was somehow adjusted to bring about inclusion of 5 wt% Au NEs into the Polymer. Followed by 1 hour stirring for homogenization, the resultant solution was cast on glass substrates, and then left to be air dried at room temperature overnight. For a second time, the samples were dried in a vacuum oven for 1 hour at 80 °C to remove the residual solvent. The prepared nanocomposites were subsequently characterized without further purification. The compliance of theoretical and practical filling factors was further proved via standardized EDX measurements. A few of the uniform composites with different filling factors were used as standards for EDX measurements (data not shown here). Subsequently, the intensity of Au peaks in the nanocomposites was correlated to the filling factor based on the previously determined calibration data.

After drying, the cast films’ thickness was measured by a stylus profilometer (Dektak 8000). To be considered in next characterizations, this value was about 600 nm for the optical measurements and gas sensing test. Whereas, it was about 1400 nm for the electrical measurements.

The structure and morphology of the samples were characterized by means of transmission electron microscopy (TEM) (Tecnai F30 G^2^ at 300 kV).

To measure electrical properties of the nanocomposites, a gold contact electrode was deposited on the surface of the films by a sputter coater (SCD 050, Balzers). The gold was sputtered and deposited onto the film surface for 2 minutes through a shadow mask with a middle gap. To create a contact thickness of 24 nm, gold was sputtered with 30 mA current under an operating pressure of 3.6 × 10^−2^ mbar. To avoid scratching the film’s surface during the measurements, a little silver paste was deposited on the gold contact electrode. The electrical conductivity was measured through the “two point probe” method using a picoamperemeter (Keithley 2400). For this measurement, a voltage of up to 6 volt was applied.

The optical properties of the samples were studied by UV-vis/IR spectrometry (Lambda 900). In order to eliminate the interference of the glass substrate, its transmittance value was subtracted from those of the measured samples.

To investigate the effect of addition of the Au NEs on thermal properties of the conductive films, Thermogravimetric analysis (TGA) were performed. This characterization was carried out with a thermogravimetric analyzer of Netzsch 209 TG. TGA analysis was performed at 20–500 °C with a heating rate of 20 °C/min under Nitrogen. The decomposition temperature (T_d_) was defined as the temperature at 5% weight loss.

To measure gas sensing property, the pristine and nanocomposite samples were exposed to the VOCs of ethanol (polar) and toluene (non-polar). Any variation in the electrical resistivity of the samples at room temperature was recorded in a given time duration through a programmable electrometer (Keithley 6517B). During the measurement, upon reaching to a steady and stable electrical resistance, the injection of VOC gas was turned off and the electrical resistance’s recovery was observed. The gas sensitivity of the samples is expressed as the normalized resistivity calculated by the following equation (7):


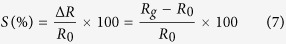


where *R*_0_ is the resistance at onset of the experiment and *R*_g_ is the resistance measured upon exposure to VOC.

## Additional Information

**How to cite this article**: Khalil, R. *et al*. A shape tailored gold-conductive polymer nanocomposite as a transparent electrode with extraordinary insensitivity to volatile organic compounds (VOCs). *Sci. Rep.* **6**, 33895; doi: 10.1038/srep33895 (2016).

## Figures and Tables

**Table 1:** Constants of *I-V* characteristics and electrical conductivity of the samples.

Sample	B	A (×10^−4^) (Ω^−1^)	σ (S/cm)
PEDOT-PSS	1.06	3.3	5.8
Au NS/PEDOT:PSS	1.01	5.6	12.2
Au NR/PEDOT:PSS	1.2	8.3	15.2

**Table 2:** Thermal properties of the PEDOT:PSS samples as pristine and nanocomposites.

Sample	Thermal decomposition temperature (°C)	Mass loss (%)
PEDOT:PSS	285	10
Au NS/PEDOT:PSS	295	6
Au NR/PEDOT:PSS	312	1

**Table 3:** The solubility parameters of the solvent and polymers and their Flory–Huggins interaction parameter, as well as the VOCs’ parameters and their concentration (ppm).

Solvent	δ_1_ (J^1/2^/cm^3/2^)	δ_2_ (J^1/2^/cm^3/2^)	v_1_^0^ (cm^3^/mol)	χ_12PSS_	χ_12PEDOT_	P (kPa, 23 °C)	D (g/cm^3^)	M (g/mol)	C_ppm_ (23 °C)
Ethanol	26.2	—	58.4	0.03	0.55	7	0.79	46.1	165.9
Toluene	18.2	—	106.2	1.98	0.41	3.4	0.87	92.1	147.6
PSS	—	25^57^	—		—	—	—	—	—
PEDOT	—	21.3^58^	—		—	—	—	—	—

**Table 4:** The effect of agglomeration on conductivity of the Au NR/PEDOT:PSS nanocomposites.

Sample	Conductivity (S/cm)
PEDOT:PSS	5.85
Agglomerated NR/PEDOT:PSS	6.16
Homogenously dispersed NR/PEDOT:PSS	15.2

## References

[b1] Lim S. K. . Fabrication of a touch sensor for flat panel displays using poly (3, 4-ethylenedioxythiophene): poly (styrene sulfonate) with dimethylsulfoxide by soft lithography. Jpn. J. Appl. Phys. 51(9R), 096501 (2012).

[b2] Kim H. K., Lee S. & Yun K. S. Capacitive tactile sensor array for touch screen application. Sens. Actuators A. 165(1), 2–7 (2011).

[b3] Madaria A. R., Kumar A. & Zhou C. Large scale, highly conductive and patterned transparent films of silver nanowires on arbitrary substrates and their application in touch screens. Nanotechnology 22(24), 245201 (2011). 21508460 10.1088/0957-4484/22/24/245201

[b4] Li D. & Guo L. J. Micron-scale organic thin film transistors with conducting polymer electrodes patterned by polymer inking and stamping. Appl. Phys. Lett. 88(6), 063513 (2006).

[b5] Park S. Y., Noh Y. H. & Lee H. H. Introduction of an interlayer between metal and semiconductor for organic thin-film transistors. Appl. Phys. Lett. 88(11), 113503 (2006).

[b6] Chen H. J. H., Chen L. C., Lien C., Chen S. R. & Ho Y. L. Nano-scale metallization of Au on flexible polyimide substrate by reversal imprint and lift-off process. Microelectron. Eng. 85(7), 1561–1567(2008).

[b7] Delozier D., Watson K., Smith J. & Connell J. Preparation and characterization of space durable polymer nanocomposite films. Compos. sci. technol. 65(5), 749–755 (2005).

[b8] Fallahzadeh A., Saghaei J. & Yousefi M. H. Effect of alcohol vapor treatment on electrical and optical properties of poly (3, 4-ethylene dioxythiophene): poly (styrene sulfonate) films for indium tin oxide-free organic light-emitting diodes. Appl. Surf. Sci. 320, 895–900 (2014).

[b9] Palumbiny C. M. . The crystallization of PEDOT: PSS polymeric electrodes probed *in situ* during printing. Adv. Mater. 27(22), 3391–3397 (2015). 25903292 10.1002/adma.201500315

[b10] Kirchmeyer S. & Reuter K. Scientific importance, properties and growing applications of poly(3,4-ethylenedioxythiophene). J. Mater. Chem. 15(21), 2077–2088 (2005).

[b11] Groenendaal L., Jonas F., Freitag D., Pielartzik H.& Reynolds J. R. poly (3, 4‐ethylenedioxythiophene) and its derivatives: past, present, and future. Adv. Mater. 12 **(7)**, 481–494 (2000).

[b12] Jang J., Chang M. & Yoon H. Chemical sensors based on highly conductive poly (3, 4‐ethylenedioxythiophene) nanorods. Adv. Mater. 17(13), 1616–1620 (2005).

[b13] Choi J., Lee J., Choi J., Jung D. & Shim S. E. Electrospun PEDOT: PSS/PVP nanofibers as the chemiresistor in chemical vapour sensing. Synth. Met. 160(13), 1415–1421 (2010).

[b14] Luo C. & Chakraborty A. Effects of dimensions on the sensitivity of a conducting polymer microwire sensor. Microelectron. J. 40(6), 912–920 (2009).

[b15] Kuş M. & Okur S. Electrical characterization of PEDOT: PSS beyond humidity saturation. Sens. Actuators B 143(1), 177–181 (2009).

[b16] Lin C. Y., Chen J. G., Hu C. W., Tunney J. J. & Ho K. C. Using a PEDOT: PSS modified electrode for detecting nitric oxide gas. Sens. Actuators B 140(2), 402–406 (2009).

[b17] Badhulika S., Myung N. V. & Mulchandani A. Conducting polymer coated single-walled carbon nanotube gas sensors for the detection of volatile organic compounds. Talanta 123, 109–114 (2014). 24725871 10.1016/j.talanta.2014.02.005

[b18] Choi J., Park D. W. & Shim S. E. Electrospun PEDOT: PSS/carbon nanotubes/PVP nanofibers as chemiresistors for aromatic volatile organic compounds. Synth. Met. 162(17), 1513–1518 (2012).

[b19] Zampetti E., Macagnano A., Pantalei S. & Bearzotti A. PEDOT: PSS coated titania nanofibers for NO_2_ detection: study of humidity effects. Sens. Actuators B 179, 69–73 (2013).

[b20] Yin K. & Zhu Z. “One-pot” synthesis, characterization, and NH_3_ sensing of Pd/PEDOT:PSS nanocomposite. Synth. Met. 160(9), 1115–1118 (2010).

[b21] Elbahri M. . An omnidirectional transparent conducting‐metal‐based plasmonic nanocomposite. Adv. Mater. 23(17), 1993–1997 (2011). 21445932 10.1002/adma.201003811

[b22] Hedayati M. K. . Design of a perfect black absorber at visible frequencies using plasmonic metamaterials. Adv. Mater. 23(45), 5410–5414 (2011). 21997378 10.1002/adma.201102646

[b23] Faupel F., Zaporojtchenko V., Strunskus T. & Elbahri M. Metal‐polymer nanocomposites for functional applications. Adv. Eng. Mater. 12(12), 1177–1190 (2010).

[b24] Beyene H. . Preparation and plasmonic properties of polymer-based composites containing Ag–Au alloy nanoparticles produced by vapor phase co-deposition. J. Mater. Sci. 45(21), 5865–5871 (2010).

[b25] Hedayati M. K., Faupel F. & Elbahri M. Tunable broadband plasmonic perfect absorber at visible frequency. Appl. Phys. A 109(4), 769–773 (2012).

[b26] Homaeigohar S. & Elbahri M. Nanocomposite electrospun nanofiber membranes for environmental remediation. Materials 7(2), 1017–1045 (2014). 10.3390/ma7021017 PMC5453108 28788497

[b27] Homaeigohar S. S. & Elbahri M. Novel compaction resistant and ductile nanocomposite nanofibrous microfiltration membranes. J. Colloid Interface Sci. 372(1), 6–15 (2012). 22304933 10.1016/j.jcis.2012.01.012

[b28] Elbahri M. . Smart metal-polymer bionanocomposites as omnidirectional plasmonic black absorbers formed by nanofluid filtration. Adv. Funct. Mater. 22(22), 4771–4777 (2012).

[b29] Homaeigohar S., Mahdavi H. & Elbahri M. Extraordinarily water permeable sol gel formed nanocomposite nanofibrous membranes. J. Colloid Interface Sci. 366, 51–56 (2012). 21999961 10.1016/j.jcis.2011.09.042

[b30] De S. . Transparent, flexible, and highly conductive thin films based on polymer− nanotube composites. Acs Nano 3(3), 714–720 (2009). 19227998 10.1021/nn800858w

[b31] Aldo J. Transparent and conductive thin films of graphene/polyaniline nanocomposites prepared through interfacial polymerization. Chem. Commun. 47(9), 2592–2594 (2011). 10.1039/c0cc04304d 21173962

[b32] Elbahri M. . Photoswitchable molecular dipole antennas with tailored coherent coupling in glassy composite. Light: Sci. Appl. 4(7), e316 (2015).

[b33] Yuan Q., Kloczkowski A., Mark J. & Sharaf M. Simulations on the reinforcement of poly (dimethylsiloxane) elastomers by randomly distributed filler particles. J. Polym. Sci. Part B 34(9), 1647–1657 (1996).

[b34] Gersappe D. Molecular mechanisms of failure in polymer nanocomposites. Phys. Rev. Lett. 89(5), 058301 (2002). 12144469 10.1103/PhysRevLett.89.058301

[b35] Liu J. . Polymer–nanoparticle interfacial behavior revisited: a molecular dynamics study. Phys. Chem. Chem. Phys. 13(28), 13058–13069 (2011). 21687850 10.1039/c0cp02952a

[b36] Karatrantos A., Clarke N., Composto R. J. & Winey K. I. Entanglements in polymer nanocomposites containing spherical nanoparticles. Soft Matter 12, 2567–2574 (2016). 26853774 10.1039/c5sm02010g

[b37] Masnada E., Merabia S., Couty M. & Barrat J. L. Entanglement-induced reinforcement in polymer nanocomposites. Soft Matter 9(44), 10532–10544 (2013).

[b38] Link S., Mohamed M. & El-Sayed M. Simulation of the optical absorption spectra of gold nanorods as a function of their aspect ratio and the effect of the medium dielectric constant. J. Phys. Chem. B 103(16), 3073–3077 (1999).

[b39] Haes A. J., Zou S., Schatz G. C. & Van Duyne R. P. A Nanoscale optical biosensor: the long range distance dependence of the localized surface plasmon resonance of noble metal nanoparticles. J. Phys. Chem. B 108(1), 109–116 (2004).

[b40] Chandran S. P., Chaudhary M., Pasricha R., Ahmad A. & Sastry M. Synthesis of gold nanotriangles and silver nanoparticles using aloevera plant extract. Biotechnol. Prog. 22(2), 577–583 (2006). 16599579 10.1021/bp0501423

[b41] Shankar S. S. . Biological synthesis of triangular gold nanoprisms. Nat. Mater. 3(7), 482–488 (2004). 15208703 10.1038/nmat1152

[b42] Shankar S. S., Rai A., Ahmad A. & Sastry M. Controlling the optical properties of lemongrass extract synthesized gold nanotriangles and potential application in infrared-absorbing optical coatings. Chem. Mater. 17(3), 566–572 (2005).

[b43] Link S. & El-Sayed M. A. Optical properties and ultrafast dynamics of metallic nanocrystals. Annu. Rev. Phys. Chem. 54(1), 331–366 (2003). 12626731 10.1146/annurev.physchem.54.011002.103759

[b44] Zhang J., Feng S. & Wang X. DC current voltage characteristics of silicone rubber filled with conductive carbon black. J. Appl. Polym. Sci. 94(2), 587–592 (2004).

[b45] Sichel E. K. Carbon Black- Polymer Composites (Marel Dekker, INC: New York, USA, : 1982).

[b46] Kim B., Park D., Joo J., Yu S. & Lee S. Synthesis, characteristics, and field emission of doped and de-doped polypyrrole, polyaniline, poly (3, 4-ethylenedioxythiophene) nanotubes and nanowires. Synth. Met. 150(3), 279–284 (2005).

[b47] Afzal A. B., Akhtar M. J., Nadeem M. & Hassan M. Investigation of structural and electrical properties of polyaniline/gold nanocomposites. J. Phys. Chem. C 113(40), 17560–17565 (2009).

[b48] Javadi H. . Charge transport in the “emeraldine” form of polyaniline. Synth. Met. 29(1), 409–416 (1989). 10.1103/physrevb.39.3579 9948674

[b49] Bengoechea M. R., Aliev F. M. & Pinto N. J. Effects of confinement on the phase separation in emeraldine base polyaniline cast from 1-methyl-2-pyrrolidinone studied via dielectric spectroscopy. J. Phys.: Condens. Matter 14(45), 11769 (2002).

[b50] Seekaew Y. . Low-cost and flexible printed graphene–PEDOT:PSS gas sensor for ammonia detection. Org. Electron. 15(11), 2971–2981 (2014).

[b51] Dehsari H. S. . Copper (ii) phthalocyanine supported on a three-dimensional nitrogen-doped graphene/PEDOT-PSS nanocomposite as a highly selective and sensitive sensor for ammonia detection at room temperature. RSC Advances 5(97), 79729–79737 (2015).

[b52] Zampetti E. . A high sensitive NO_2_ gas sensor based on PEDOT–PSS/TiO_2_ nanofibres. Sens. Actuators, B 176, 390–398 (2013).

[b53] Taccola S. . Characterization of free-standing PEDOT:PSS/iron oxide nanoparticle composite thin films and application as conformable humidity sensors. ACS Appl. Mater. Interfaces 5(13), 6324–6332 (2013). 23802632 10.1021/am4013775

[b54] Lin C. Y., Chen J. G., Hu C. W., Tunney J. J. & Ho K. C. Using a PEDOT:PSS modified electrode for detecting nitric oxide gas. Sens. Actuators B 140(2), 402–406 (2009).

[b55] Yeo J. S. . Significant vertical phase separation in solvent-vapor-annealed poly(3,4-ethylenedioxythiophene):poly(styrene sulfonate) composite films leading to better conductivity and work function for high-performance indium tin oxide-free optoelectronics. ACS Appl. Mater. Interfaces 4(5), 2551–2560 (2012). 22489686 10.1021/am300231v

[b56] Walheim S., Böltau M., Mlynek J., Krausch G.& Steiner U. Structure formation via polymer demixing in spin-cast films. Macromolecules 30(17), 4995–5003 (1997).

[b57] Kaevand T., Öpik A. & Lille Ü. A mesoscale simulation of the morphology of the PEDT/PSS complex in the water dispersion and thin film: the use of the MesoDyn simulation code. In Advances in Computer and Information Sciences and Engineering 540–546 (Springer, 2008).

[b58] Li Y., Feng Y. & Feng W. The synthesis of poly (3, 4-ethylenedioxythiophene) micro/nano-spheres by the demulsifying treatment. Synth. Met. 162(9), 781–787 (2012).

[b59] Bi N. . Analysis of immunoreaction with localized surface plasmon resonance biosensor. Spectrochim. Acta Part A 75(3), 1163–1167 (2010). 10.1016/j.saa.2009.12.084 20079682

[b60] Nikoobakht B. & El-Sayed M. A. Preparation and growth mechanism of gold nanorods (NRs) using seed-mediated growth method. Chem. Mater. 15(10), 1957–1962 (2013).

